# Improvement in clinical outcome and infection control using molecular diagnostic techniques for early detection of MDR tuberculous spondylitis: a multicenter retrospective study

**DOI:** 10.1038/emi.2017.83

**Published:** 2017-11-08

**Authors:** Wenjie Wu, Jingtong Lyu, Peng Cheng, Yuan Cheng, Zehua Zhang, Litao Li, Yonghong Zheng, Jianzhong Xu

**Affiliations:** 1 Department of Orthopaedics, Southwest Hospital, Third Military Medical University, Chongqing, China; 2 Department of Orthopaedics, Yulin People’s Hospital, Yu Lin, China; 3 Department of Orthopaedics, The People’s Liberation Army No. 309 Hospital, Beijing, China; 4 Department of Orthopaedics, Xi’an Jiaotong University Affiliated Honghui Hospital, Xi’an, China

**Keywords:** clinical outcomes, multidrug-resistant, molecular diagnosis, tuberculous spondylitis

## Abstract

There has been limited research on the therapeutic efficacy of molecular diagnosis of spinal tuberculosis. We attempted to determine whether the utilization of molecular diagnosis to detect multidrug-resistant spinal tuberculosis can improve clinical outcomes. A multicenter retrospective study was conducted from February 2009 to June 2015. Ninety-two consecutive culture-confirmed multidrug-resistant tuberculosis (MDR-TB) patients with spinal tuberculosis who were diagnosed clinically and by imaging were enrolled in the study. The initial time to treatment for MDR-TB, the method of infection control, the erythrocyte sedimentation rate (ESR) and the occurrence of complications in patients who were diagnosed using molecular methods were compared with those of patients diagnosed using standard culture and drug susceptibility test methods. Of 92 MDR-TB patients with spinal tuberculosis, 41 (45%) were diagnosed by standard culture and drug susceptibility test methods (Group A), and 51 (55%) were diagnosed following implementation of detection using molecular diagnosis (Group B). The patients in Group B began the rational use of second-line drugs earlier than patients in Group A (5 days vs 73 days, *P*<0.05). Among patients who were admitted to a general tuberculosis ward, those in Group B spent less time in the ward than those in Group A (4 days vs 33 days, *P*<0.05). At the one-month follow-up, the ESR was significantly lower in Group B. In patients who completed 6 months of follow-up (*n*=92), the incidence of complications was significantly lower in Group B. The use of molecular diagnosis resulted in noteworthy clinical advances, including earlier initiation of MDR-TB treatment, improved infection control, better clinical outcome, a more rapid decrease in ESR and fewer complications.

## INTRODUCTION

The number of new tuberculosis (TB) cases in China was 980 000 in 2013; multidrug-resistant tuberculosis (MDR-TB) accounted for ~5.7% of these cases, and the proportion of MDR-TB in patients undergoing retreatment was as high as 26%. There remains a large gap in the detection and treatment of global MDR-TB. China is currently ranked first among the 27 countries with the highest burden of MDR-TB and extensively drug-resistant tuberculosis in the world.^1^ Spinal TB, almost all cases of which originate from pulmonary TB, is one of the most common extrapulmonary TB pathologies. It can result in serious complications and is also subject to the challenge of drug resistance.

Anti-TB drug regimens are the cornerstone of spinal TB treatment. Effective drug regimens are fundamentally required to kill *Mycobacterium tuberculosis* and cure the disease. Early diagnosis of drug resistance and drug susceptibility testing (DST)-guided individualized chemotherapy are crucial for the optimal management of this disease. However, conventional DST for *M. tuberculosis* still relies on culture of the bacilli and requires a minimum of several weeks.^2^ The use of ineffective anti-TB regimens during the testing period may result in acquired drug resistance and local recurrence.^3^ Moreover, DST for TB spondylitis is not performed routinely in most resource-poor hospitals in China due to biosafety concerns and inadequate infrastructure, and this presents a major hindrance to the treatment of the disease. Therefore, rapid, accurate detection and early diagnosis are necessary for the successful management of MDR-TB in China.^4^

In recent years, a number of molecular DST kits, such as INNO-LiPA, Genotype MDR-TBplus, DNA microarrays and Xpert MTB/RIF, have been developed for the detection of mutations associated with resistance to Rifampin (RMP) and Isoniazide (INH).^5, 6, 7, 8, 9, 10^ A number of studies have verified that these molecular testing methods are extremely effective in detecting TB and relevant drug resistance, particularly in acid-fast bacilli smear-positive samples.^11, 12^ Nevertheless, there is little relevant information on the clinical outcomes of patients who have undergone rapid molecular diagnosis of MDR-TB, especially spinal TB.

**Figure 1:**
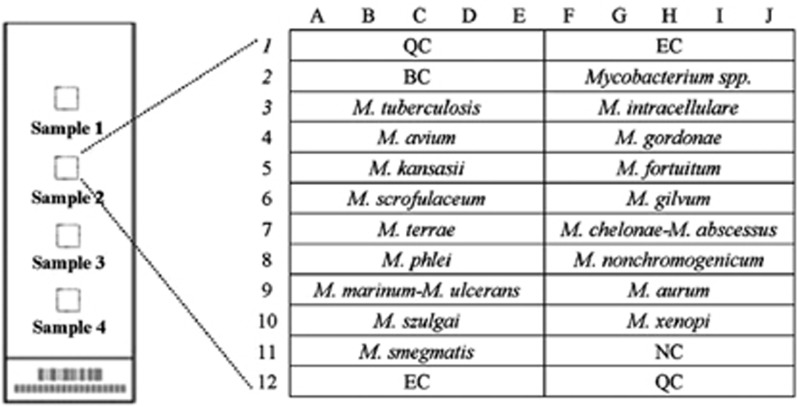
Schematic diagram of the DNA probe array for different Mycobacterium species. Seventeen oligonucleotide probes that hybridize to species-specific sequence regions of the 16S *rRNA* gene were chosen for identification of different Mycobacterium species. All probes were immobilized horizontally five times.

**Figure 2:**
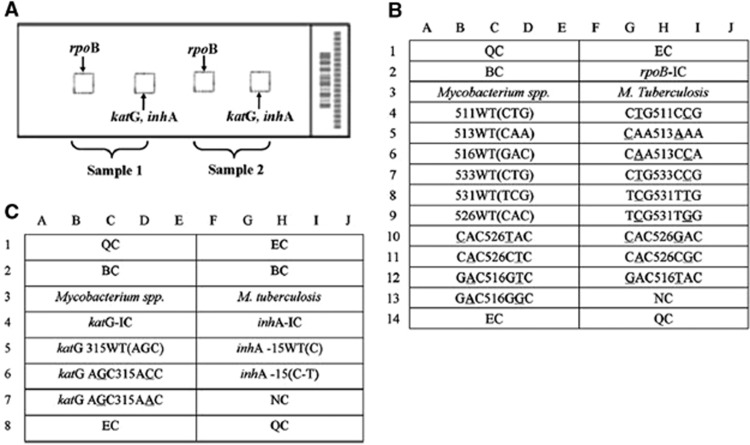
Schematic diagram of the DNA probe array for *rpoB, katG and inhA* detection. (**A**) The biochip contains two microarrays, and two specimens can be analyzed in parallel; in each array, one subarray is for RMP, and the other is for INH. (**B**) Six *rpoB* wild-type probes and thirteen mutation-type probes were designed for the detection of RMP resistance. (**C**) For the detection of INH resistance, one probe covers the wild-type codon 315 of *katG* and two mutation-type probes for the same region, and one wild-type probe and one mutation-type probe are used to detect the *inhA* promoter region. All probes were immobilized horizontally five times. QC, quality controls; EC, external controls; BC, blank controls; NC, negative controls; IC, internal controls; WT, wild type.

**Figure 3:**
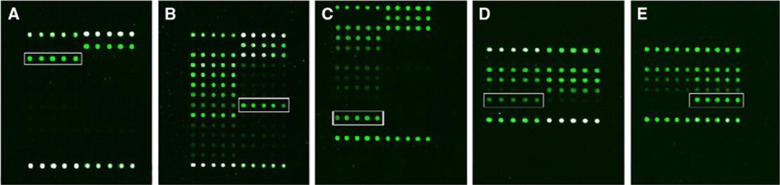
Hybridization pattern of spinal tuberculosis specimens obtained with the DNA probe. (**A**) Hybridization patterns on the gene chip used to identify *M. tuberculosis* strains (white frame). (**B**) RMP resistance detected by the presence of the *rpoB* 531 (TCG->TTG) mutation; the hybridization signal generated by probe TCG531TTG (solid rectangle) is more intense than that generated by the corresponding wild-type probe (dashed rectangle). (**C**) RMP resistance detected by the presence of the *rpoB* 516 (GAC->GTC) mutation; the hybridization signal generated by probe GAC516GTC (solid rectangle) is more intense than that generated by the corresponding wild-type probe (dashed rectangle). (**D** and **E**) INH resistance tested by the presence of the *katG* 315 (AGC->ACC) mutation; the hybridization signals generated by the *katG* probe AGC315ACC (solid rectangles) are more intense than those generated by the corresponding wild-type probe (dashed rectangles). (**E**) INH resistance detected by the presence of the *inhA*-15 C-T mutation; the hybridization signal generated by the *inhA* 15C→T probe (solid rectangle is higher than that generated by the corresponding wild-type probe (dashed rectangle). (Figures 1, 2, 3 were from a previous publication by our team that appeared in BMC Infectious Diseases 2012).

Previous research in this area has resulted in the development of a TB DST chip. The gene chip is a DNA microarray chip, the function of which is based on the principle of hybridization of DNA to oligonucleotides immobilized on the chip surface. To create the chip used in our study, oligonucleotide probes were printed onto OPAldehydeSlidealdehyde-activated slides (CapitalBio Corporation, Beijing, China) and were covalently immobilized on the slides via amino groups at their 5′ ends. Each array contained 16 oligonucleotide probes designed (CapitalBio Corporation, Beijing, China) to detect mutations of *rpoB* (codons 531, 526, 513, 516, 511 and 533), *inhA* (nucleotide 15 within the promoter) and *katG* (codon 315). In addition, 17 oligonucleotide probes for several species-specific sequence regions of the 16S *rRNA* gene were chosen for identification of different Mycobacterium species (Figures 1, 2, 3).^13^

The molecular diagnosis methods used in three of the hospitals included Genotype MDR-TBplus and Xpert MTB/RIF. We performed these tests according to the standard test procedures for each.^2, 4, 11^

In this research, we attempted to clarify whether the use of molecular diagnostic methods (CapitalBio™ DNA Microarray assay, Genotype MDR-TBplus and Xpert MTB/RIF) to detect multidrug-resistant spinal TB can improve clinical outcomes.

## MATERIALS AND METHODS

### Study patients and research design

We conducted a multicenter retrospective study of patients with MDR spinal TB in four hospitals.^14^ All of the study participants were diagnosed using culture methods for MDR, and the diagnosis was confirmed by conventional DST. Forty-one patients were diagnosed solely by conventional culture and DST methods, and 51 patients were diagnosed following implementation of detection using molecular diagnosis. These hospitals serve areas in which there is a high incidence of TB. A sequential series of patients were initially identified using molecular diagnosis; these patients were then diagnosed clinically and by imaging and compared with similar patients who had been identified by conventional culture and DST. We reviewed the ESR at 1, 3 and 6 months. The study was approved by the Ethics Committee of the Southwest Hospital.

### Treatment

In accordance with the recommendations of the MDR-TB treatment association in China, a standard chemotherapy regimen was implemented for each patient before his or her second-line drug sensitivity test results were available. After receiving the final DST data, an individualized therapeutic regimen was implemented for each patient in accordance with the test results and as recommended by the World Health Organization (WHO).^1^ The treatments were designed to include 4–5 drugs to which the patient’s *M. tuberculosis* isolate was susceptible. All therapeutic protocols included fluoroquinolone, an injectable agent (streptomycin or aminoglycosides), isoniazide and pyrazinamide. All TB patients received therapy through directly observed treatment. On beginning MDR-TB therapy, patients were started on primary treatment and were not required to remain in the hospital until they had acquired good body condition and normal laboratory indicators. Whether or not patients with drug-susceptible TB were hospitalized in a center for patients with infectious disease depended on the physician’s judgment. Patients whose diagnosis was uncertain or who were awaiting DST results were commonly admitted to a ward housing patients with drug-susceptible infections. Once a patient was diagnosed with MDR-TB, the patient was required to enter an isolation ward for patients with drug-resistant infections. Patients who required individualized surgical procedures due to their general condition underwent focal debridement, fusion and instrumentation. The absolute surgical indications included severe deformity, spinal instability and nerve dysfunction. Many patients in whom spinal lesions are reduced by drug therapy can be treated conservatively or with percutaneous catheter drainage and local chemotherapy.

### Statistical analysis

The *χ*^2^ test or Fisher’s exact test was used to examine categorical variables; for continuous variables, the *t*-test was applied. *P*-values of <0.05 were considered to indicate statistical significance.

## RESULTS

### Demographic and clinical characteristics

We enrolled a total of 92 patients with MDR-TB. Approximately 89% of the enrolled patients had suffered TB previously (Table 1). In this research, the results obtained using molecular diagnosis were for the most part consistent with the results obtained by conventional culture+DST. Only four patients who were diagnosed using molecular methods displayed resistance to two types of drugs. The culture method verified resistance to one type of drug. Despite the differences between individual samples, these differences did not affect the use of second-line drugs. Only two patients whose diagnoses were made using the chip hybridization method harbored sensitive isolates. The culture method verified RFP resistance in these patients. These two patients were treated with first-line drug regimens for 65 and 73 days, respectively, until the DST showed resistance.

### Treatment outcomes

All patients (*n*=92) received a first-line drug regimen for some time. Among patients receiving first-line anti-TB drugs, the duration of therapy was 22 days in Group B and 154 days in Group A (*P*<0.05). Relative to Group B, the start of second-line pharmacotherapy in sufferers in Group A was delayed (5 vs 73 days, *P*<0.05) (Table 2).

### Specialized measures used to control contagion

All patients diagnosed using molecular technology were eventually hospitalized for MDR-TB therapy and placed in drug resistance sickrooms. In contrast, patients diagnosed by standard culture and drug susceptibility test methods were placed in sickrooms designated for patients with drug-susceptible infections.

Of the 92 inpatients with MDR-TB, the patients who were enrolled in Group A spent longer in sickrooms designated for patients with drug-susceptible infections (33 vs 4 days, *P*<0.05) and were likely to spend extended periods in hospital wards (33 vs 21 days, *P*<0.05) compared with the patients in Group B. Thirty-eight MDR-TB patients started extramural hospital treatment using first-line drugs before their drug susceptibility test results were known. Of these 38 patients, the mean time until hospitalization and starting second-line drug pharmacotherapy was 73 days in Group B compared with 126 days in Group A (*P*<0.05) (Table 3).

### Laboratory index

At the third follow-up, the ESR was decreased; a greater reduction occurred in Group B than in Group A (67% vs 48%, *P*<0.05). At the sixth follow-up, there was no obvious difference in ESR between Groups A and B (*P*>0.05).

### Complications

All patients received anti-TB treatment, and some patients (33%) had complications. Gastrointestinal symptoms were the most common (70%), followed by sinus problems (30%), hepatotoxicity (20%) and delayed healing of incisions (17%). No patients experienced neurological symptoms or hearing impairment. The complication rate was significantly lower in Group B than in Group A (10% vs 61%, *P*<0.05) (Table 4). In Group A, seven patients had sinus problems, and four patients experienced delayed wound healing. In Group B, two patients had sinus problems and one patient experienced delayed wound healing. These complications were resolved by symptomatic treatment.

## DISCUSSION

Patients with spinal TB are often seen by physicians in general hospitals, in which there is a lack of protective measures and limited experience in diagnosis, making the treatment and control of TB difficult. The ineffective anti-TB regimens that are often prescribed under these conditions may cause acquired drug resistance and local recurrence. The application of molecular diagnosis technology resulted in an apparent improvement in the clinical therapeutic effect among MDR-TB patients with spondylitis in this study.

Some researchers have paid close attention to nosocomial transmission and to the cross-infection of patients with drug-susceptible TB by patients with MDR-TB.^15, 16^ Using molecular probe technology to detect TB, we are able to rapidly diagnose MDR-TB and begin the use of second-line anti-TB drugs as soon as possible. At the same time, we can control TB nidus, achieve earlier surgical treatment for spinal TB, shorten patients’ hospitalization times and improve the effects of drug treatments.

Although many articles pay close attention to the diagnostic efficiency of rapid molecular tests,^17, 18^ very few studies have determined the clinical efficacy of these tests. Only three published papers have reported the clinical effects of using probe assays to detect MDR-TB.^8, 19, 20^ Two of these studies were conducted in South Africa and assessed the starting time of MDR-TB therapy. One study reported that the time to use second-line anti-TB drugs was reduced from 78 to 62 days after use of the MTBDRplus method, and the other reported a similar decrease from 80 to 55 days. Both studies emphasized that clinical and experimental operating problems delayed the delivery and consequently the interpretation of research results. A very recent paper evaluating the clinical effects of using the Xpert method reported the clinical effects of using the method to detect MDR-TB in much more detail^8^ and noted a marked reduction in the time to initiation of second-line drugs. In our research, the experimenter rapidly communicated the data obtained through molecular diagnosis technology to physicians, and this obviously shortened the time before the initiation of treatment with second-line drugs.

Drug therapy is the basis of spinal TB treatment. Furthermore, the use of individualized, appropriate anti-TB drug therapy can significantly improve the effects of surgery, especially for patients with MDR-TB of the spine. Optimal management of MDR-TB relies on the early detection of the disease in such patients.^15^ Our previous studies have confirmed that the gene chip is a feasible and accurate tool for the species identification of *M. tuberculosis* and for the diagnosis of MDR-TB.^13^ In this research, we observed improved therapeutic effects in patients in whom the gene chip was used for diagnosis. For example, local symptoms can be eased significantly or even disappear with the use of medication, thus avoiding surgery. Almost all of these cases occurred in Group B, and Group B was superior to Group A in clinical outcome. In addition, more patients in Group B accessed surgical treatment at an earlier time.

Preventing the spread of TB infection in hospitals is a problem that has been too long ignored in strategies for infection control for MDR-TB, especially in low- and middle-income developing countries.^19, 20^ The main reason for this is the lack of effective detection and treatment of MDR-TB. Our research shows that the use of accurate and rapid molecular diagnostic technology to detect MDR-TB can improve clinical therapeutic effects and markedly reduce the length of hospital stay of patients with MDR-TB in drug-susceptible wards. At the same time, it is essential to reduce the chances of nosocomial infection by MDR-TB and to improve the effects of surgery for patients with spinal TB. We also observed that accurate and rapid detection of MDR-TB can dramatically shorten the time that patients use ill-suited first-line drug treatments outside the hospital setting and that it can effectively prevent the transmission of MDR-TB.

Since China formulated its national plan for the prevention and cure of TB in 1991, there has been a great deal of progress in TB control. At present, the standard TB treatment in China mainly uses four kinds of first-line drugs and other anti-TB chemotherapeutic drugs. However, all drugs can cause adverse reactions of varying degrees and frequencies. When such reactions occur, the patient’s adherence to treatment is often reduced, and this directly affects the prevention and control of TB. The incidence of adverse reactions to anti-TB drugs has increased significantly over time. In this research, we analyzed the adverse drug reactions that occurred in two groups of patients before using second-line drug treatment. Overall, gastrointestinal symptoms and hepatotoxicity occurred most frequently, and no neurological symptoms or hearing impairment occurred. The rate of adverse drug reactions was markedly lower in the molecular diagnosis group than in the conventionally diagnosed group. We consider that this may have been due to the earlier confirmed drug resistance status of these patients and the resulting decrease in the duration of their treatment with first-line drugs. A shortcoming of this study was that it did not analyze the participants’ adverse reactions to second-line drugs. In the future, we will conduct an in-depth study of the adverse reactions to all prescribed drugs; thereby, we expect to achieve more comprehensive results. In the current study, the occurrence of sinus problems and delayed wound healing in 3 and 2 patients, respectively, in group A may be related to the delay in drug resistance detection and/or to the delay in adjusting the chemotherapy regimen in these patients.

Our study has some limitations. First, it is a retrospective study with a small sample size. Second, it is uncertain whether our laboratory findings can be extrapolated to patients in other urban or even rural areas. However, the results of this research have important value as a guideline for other studies.

## CONCLUSION

In summary, molecular diagnostic technology offers a rapid and accurate diagnostic tool for *M. tuberculosis* identification and resistance testing. Using this tool, we can rapidly analyze the drug resistance of spinal TB specimens, increase clinical efficacy and decrease delays in diagnosis, thereby reducing the need to build large numbers of advanced biosafety facilities. However, because infrastructure and trained professionals are required for the use of this technology, it is currently available only in a limited number of reference laboratories, which reduces its clinical utility in poverty-stricken zones. Thus, more integral and fully automatic gene detection systems should be developed. In addition, additional genetic mutations related to first-line and second-line drug resistance should be considered for incorporation into future gene detection systems because of the dissemination of *M. tuberculosis* strains with resistance to second-line drugs. Furthermore, a large sample prospective cohort study with long-term follow-up should be designed and conducted to further assess the efficiency of methods for the molecular diagnosis of spinal TB.

## Figures and Tables

**Table 1:** Demographic and clinical characteristics of patients tested using conventional culture methods or molecular technology

**Characteristic**	**Overall** ***n*****=92 (%)**	**Culture methods 41 (%)**	**Molecular technology 51 (%)**
Mean age (range)	33.5 (2–63)	34.5 (8–63)	32.7 (2–55)
Male	56 (61)	26 (63)	30 (59)
Countryside	68 (70)	31 (46)	37 (54)
History of tuberculosis	82 (89)	36 (44)	46 (56)
*Prior tuberculosis treatment*			
None	54 (59)	15 (37)	39 (76)*
First-time treatment	38 (41)	26 (63)	12 (24)*

*P<0.05.

**Table 2:** Therapeutic outcome of MDR-TB patients diagnosed using conventional culture and molecular diagnosis technology (*n*=92)

**Characteristic**	**Overall** ***n*****=92 (%)**	**Culture methods 41 (%)**	**Molecular diagnosis 51 (%)**
Treatment with first-line drug regimen (%)	92 (100)	41 (100)	51 (100)
Mean days on first-line drug (range) (*n*=92)	81 (14–434)	154 (73–434)	22 (14–36)
Mean days until starting MDR-TB treatment using second-line drug (range) (*n*=92)	44 (6–85)	73 (54–102)	5 (2–6)
			
*Required surgery (%)*	87 (95)	41 (100)	46 (90)
Recovered without need for surgery	5	0	5

**Table 3:** Specialized control measures for contagion

**Characteristic**	**Overall** ***n*****=92 (%)**	**Culture methods 41 (%)**	**Molecular diagnosis 51 (%)**
*Infection control measures*			
Placed in drug-susceptible sickroom (%)	92 (100)	41 (100)	51 (100)
Mean days in drug-susceptible sickroom (range) (*n*=92)	17 (2–58)	33 (16–58)	4 (2–5)
Mean days until hospitalization and starting second-line drug pharmacotherapy (range) (*n*=38)	109 (30–365)	126 (30–365)	73 (30–330)
Total days of hospitalization (range) (*n*=92)	26 (10–58)	33 (16–58)	21 (10–35)
Mean days of drug treatment prior to hospitalization (range) (*n*=92)	48 (14–105)	82 (15–367)	18 (13–31)

**Table 4:** Complications (*n*=30)

**Characteristic (%)**	**Overall (92)**	**Conventional culture (41)**	**Molecular diagnosis (51)**
*Complications (n=30)*			
Gastrointestinal symptoms	21 (23)	18 (44)	3 (6)
Hepatotoxicity	6 (7)	6 (15)	0 (0)
Sinus	9 (10)	7 (17)	2 (4)
Delayed healing of incision	5 (5)	4 (10)	1 (2)
Total complications	30 (33)	25 (61)	5 (10)*

*P<0.05.
